# Comparison of Wear Resistance of Overlay Welded Layers and Thermal Sprayed Coatings in Real Conditions

**DOI:** 10.3390/ma16227215

**Published:** 2023-11-17

**Authors:** Michał Szymura, Grzegorz Gąsiorek, Artur Czupryński, Waldemar Kwaśny, Viktor Kvasnytskyi

**Affiliations:** 1 FPM S.A., Wyzwolenia Street 12C, 43-190 Mikołów, Poland; mich.szymura@gmail.com (M.S.); grzegorz.gasiorek@fpmsa.com (G.G.); 2 Department of Welding, Silesian University of Technology, Konarskiego Street 18A, 44-100 Gliwice, Poland; waldemar.kwasny@polsl.pl; 3 Igor Sikorsky Kyiv Polytechnic Institute, 37, Peremohy Ave., 03056 Kyiv, Ukraine; kvas69@ukr.net

**Keywords:** operational tests, wear, flux cored arc surfacing, thermal flame spraying, chrome cast iron, nickel alloy, vertical ball-race mill

## Abstract

Tribological tests in real conditions enable obtaining full data on the life of interacting machine parts. This article presents the results of operational tests on the elements of the support ring guidance system in a vertical ball-race mill. The guide and active armour operate under abrasive wear conditions with moderate-impact loads. The wear resistance of elements with overlay welding layers deposited with flux cored wire with a structure of high-alloy chrome cast iron and with a coating flame-sprayed with nickel-based powder was compared. The wear intensity of the overlay weld deposits was much lower than that of the sprayed coatings. The scope of this study also included the analysis of the chemical and phase composition, macro- and microscopic metallographic examinations, and the measurement of the hardness of the deposited layers and coatings.

## 1. Introduction

Coal dust used in power plants is obtained from crumble coal grains in a mill. Vertical roller mills and vertical ball-race mills are most often used for grinding hard coal. In a ball-race mill, the milling process takes place by crushing and grinding the milling material. This process takes place between the crushing ring and the grinding balls, which are pressed by the pressure ring and the stop ring located on it. The guidance system consisting of guide and armour (active and passive) is responsible for maintaining the pressure ring in the mill axis through the stop ring. The dominant wear process of the guide and active armour is abrasion occurring at moderate-impact loads. The result of wear is an increase in the gap between the surfaces of the guide and the passive armour. The lack of systematic adjustment of the gap length leads to an improper pressure of the balls on the crushing ring, increasing the vibration level of the mill and then leading to its failure as a result of damage caused to the spring pressure system (spring fracture) [1,2].

Welding technologies enable the application of abrasion-resistant layers and coatings. This resistance depends on the type of additional material used and the surfacing technology [3,4,5]. During the surfacing with arc welding process, dilution occurs, which may cause differences in the structure of the surfacing layer, and thus changes in its abrasion resistance depending on the degree of wear [6]. Thermal spray processes are characterized by the absence of melting of the base material. According to Dallaire [7], the submerged-arc surfacing Fe-Cr-C layer showed a much smaller (an over seven-fold) volume loss in the ASTM G-65 abrasion resistance test compared to an arc-sprayed coating consisting of a nickel-based alloy containing fine borides and carbides.

Laboratory tests of wear resistance enable the simulation of the wear of elements for certain specific real conditions; therefore, they are comparative tests [7,8,9,10]. Generally, abrasion resistance testing devices can be divided into three categories according to the type of abrasion: (two-body and three-body), contact kinematics (sliding, rolling, and reciprocating, impact), and contact medium (dry, wet, and slurry) [11]. Most abrasion testing equipment is designed in accordance with the prescribed standards (e.g., ISO and ASTM). Changing the design of testing devices or test parameters may lead to differences in the results obtained [11,12,13]. The study [9] on the comparison of abrasion resistance of layers deposited by flux cored arc surfacing (including Fe-Cr-Nb-C alloy) in various abrasion tests (dry-sand rubber-wheel tester and impeller–tumbler tests at low and high energy) shows that the Fe-Cr-Nb-C alloy does not wear evenly under high loads because the plastic deformation of the matrix occurs and the chromium carbides crack. The study [6] on the influence of the type of abrasive material on the abrasive wear resistance of wear plates with a high-alloy chromium cast iron overlay made by self-shielded flux cored arc surfacing in an abrasion test based on ASTM G-65 showed that the type of abrasive material (analysed materials: rounded quartz grain sand, clinker sand, and blast furnace slag) has a decisive influence on the abrasive wear resistance of surface layers. In the study [14] comparing the wear intensity of flame-sprayed NiCrBSi coatings and cast iron samples in the ball on disc wear test, it was found that the ratio of their abrasion resistance is not the same when their sliding speed and their load was changed. Abrasive wear is a complex process and, in relation to the operation of parts of machines and devices in industrial conditions, it may be impossible to develop a model on a laboratory scale due to the inability to precisely determine the wear conditions (forces, unit pressures, friction speed, type of abrasive, etc.), among other factors.

The available literature presents the results of laboratory tests of abrasion resistance of both overlay welding layers with high-alloy chromium cast iron [6,8,15,16,17,18,19] and flame-sprayed coatings with nickel-based powder [20,21,22,23,24]. However, there was no quantitative data enabling the comparison of the wear intensity of layers and coatings applied to guides and active armour in real conditions. The data obtained in this way can be directly used in industrial practice. Therefore, an attempt was made to compare the resistance to abrasive wear with moderate-impact loads of the mentioned kinematic pair with overlay welded wear-resistant layers and with thermal sprayed wear-resistant coatings.

This article presents the results of operational tests of the elements of the support ring guidance system in a vertical ball-race mill. The analysed guides and active armour were protected with a layer deposited by flux cored arc surfacing with the structure of high-alloy chrome cast iron and a flame-sprayed coating with nickel-based powder. The scope of this study also included the analysis of the chemical and phase composition, macro- and microscopic metallographic examinations, and the measurement of the hardness of the deposited layers and coatings.

## 2. Materials and Research Methods

### Materials and Their Preparation

**Figure 1:**
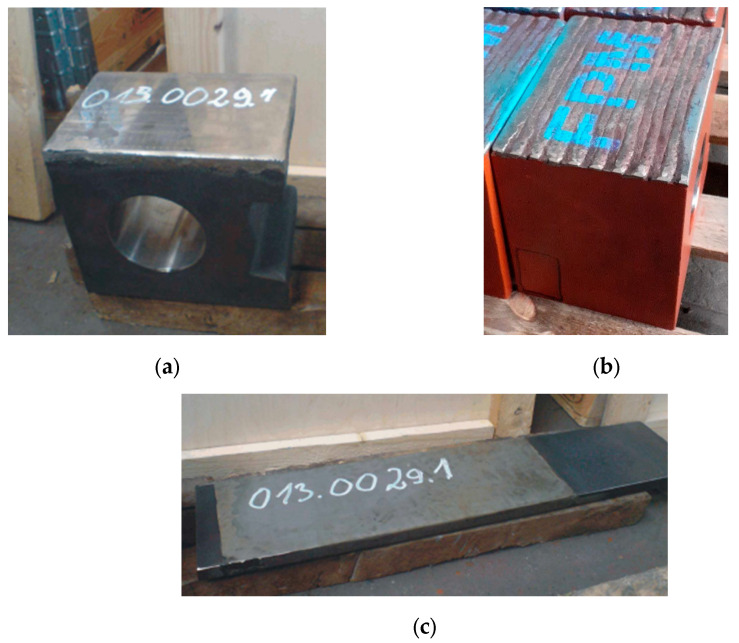
Images of (**a**) guide with a thermal sprayed coating; (**b**) guide with a flux cored arc surfaced layer; and (**c**) active armour with a thermal sprayed coating.

For operational tests, two sets (guide and active armour) of the EM-70 mill thrust ring guidance system manufactured by FPM were made. The guides are made of cast steel for general engineering uses, GS240 according to EN 10293 [25], and the active armour is made of non-alloy structural steel, S235JR according to EN 10025-2 [26]. The chemical composition and mechanical properties of these material are provided in Table 1 and Table 2. The working surfaces of the elements of the first set were surfaced with self-shielded flux cored wire SK A43-O produced by voestalpine Böhler Welding (Voestalpine AG, Linz, Austria), ensuring an overlay weld deposit of high-alloy chrome cast iron, Fe15 according to EN 14700 [27]. This wire is recommended for protecting surfaces exposed to intense abrasive wear with moderate- and high-impact load. The chemical composition and hardness of the weld deposit of the filler material are provided in Table 3. Metco 15E powder from Oerlikon Metco (OC Oerlikon Corporation AG, Pfäffikon, Schwyz, Switzerland) was used to flame spray the surface of the guide and the active armour of the second set. According to the manufacturer, coatings that are thermally sprayed with Metco 15E powder are resistant to tribological wear and corrosion. The chemical composition and hardness of the coating sprayed with this powder are presented in Table 4. Compared to the layers deposited by arc welding with self-shielded flux cored wire, coatings flame-sprayed with self-fluxing powders after melting are characterized by a much greater surface smoothness, which may contribute to an increase in the durability of the friction pair [20]. The technological parameters of deposited layers and coatings are presented in Table 5 and Table 6. Photos of elements surface-coated by welding and thermal spraying for operational tests are presented in Figure 1. For laboratory tests, elements cut from S235JR non-alloy steel sheet with dimensions of 250 × 100 × 20 mm were used as the base material.

## 3. Characteristics of the Tests

### 3.1. Operational Tests

**Figure 2:**
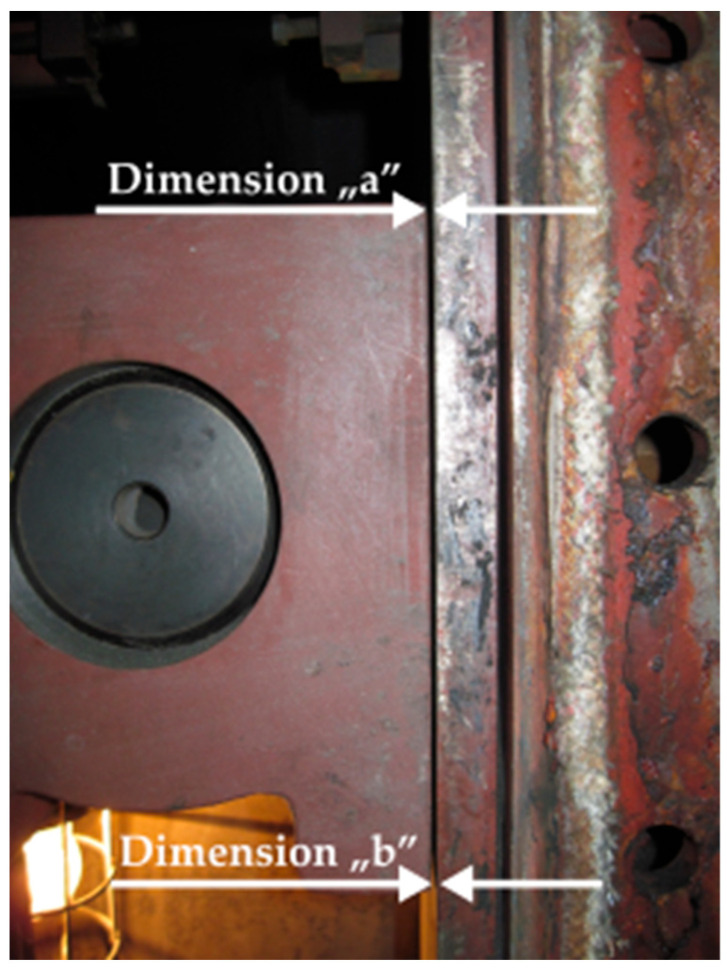
Location of a pair of measurements of the size of the gap between the guide and the passive armour.

Operational tests of two sets of support ring guidance systems were carried out in the EM-70 ball-race mill located in a combined heat and power plant powered by hard coal. The tests were performed in real conditions during the standard operation of the heat and power plant. The wear resistance of the made layers and coatings was defined as the operating time (in hours) per unit of wear (corresponding to the size of the structural gap between the guide and the passive armour). The larger value of the pair of gap width measurements in the upper and lower areas was considered as the gap size (Figure 2).

**Figure 3:**
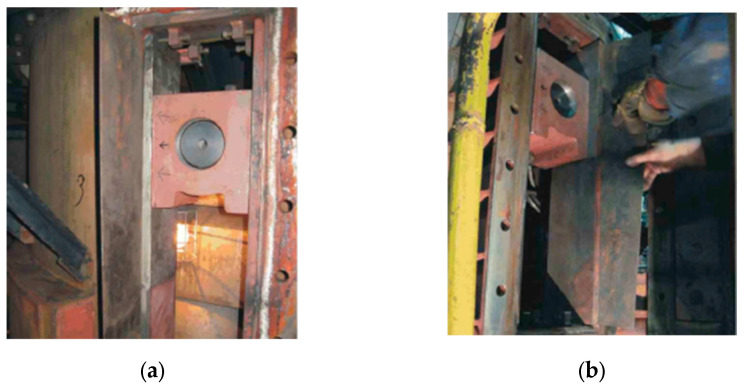
Installation of the guide and active armour with a sprayed coating in the guide chamber: (**a**) view of the installed elements and (**b**) adjustment of the gap between the guide and the passive armour.

The correct installation of the guidance system and its appropriate adjustment during the mill’s operation period requires maintaining the mentioned construction gap in the range of 2 ± 1 mm. The gap width measurements were performed during scheduled mill inspections, i.e., approximately every 700–800 h of operation. In accordance with the technical and operational documentation, in order to assemble the guide set, the guidance chamber was opened, and the previous guide and active armour were demounted. Then, the guiding set with the sprayed coating was installed, Figure 3a, and the construction gap was established, Figure 3b. Before closing, the conduction chamber was sealed. The same procedure was followed for the set with the overlay welded layer.

### 3.2. Chemical Composition Analysis

The analysis of the chemical composition in the micro-areas of the samples was performed using a Hitach S-3400N scanning electron microscope (Hitachi, Ltd., Marunouchi, Chiyoda, Tokyo, Japan) equipped with a Noran SYSTEM SIX EDS spectrometer (Thermo Fisher Scientific Inc., Waltham, MA, USA).

### 3.3. X-ray Qualitative Phase Analysis

X-ray phase analysis of the samples was performed using a JDX-7S diffractometer from JEOL (JEOL Ltd. Akishima, Tokyo, Japan). Filtered radiation from a copper X-ray tube was used. Measurements were performed in the angular range from 30° to 90° of angle with a step of 0.05° 2θ and a counting time of 5 s. Phase identification was performed based on the International Centre for Diffraction Data PDF-4+ database, year 2023.

### 3.4. Macro- and Microscopic Metallographic Examinations

In order to determine the quality of layers and coatings, macro- and microscopic metallographic examinations were performed. Macro- and microscopic metallographic examinations were carried out on transverse metallographic sections for each sample using SZX9 and GX71 light microscopes from Olympus (Olympus Corporation, Shinjuku, Tokyo, Japan). Microscopic observations at higher magnifications were performed on a Hitach S-3400N scanning electron microscope (Hitachi, Ltd., Marunouchi, Chiyoda, Tokyo, Japan). The porosity of the thermal sprayed coating was determined based on the analysis of ten microstructure images at ×200 magnification using the Special AnalySIS pro program, version JMP9 (JMP Statistical Discovery LLC, Cary, NC, USA).

### 3.5. Hardness Measurement

**Figure 4:**
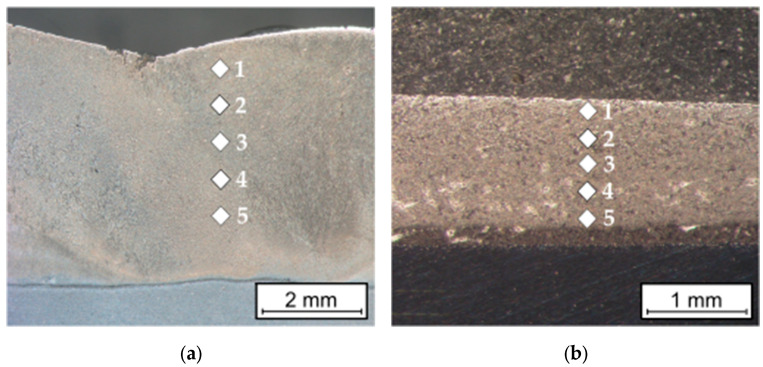
Pictorial diagram of the location of hardness measurement points on the cross-sectional surface of (**a**) the overlay welded layer and (**b**) sprayed coating.

The hardness measurements of the overlay welded layer and the sprayed coating were carried out using the Vickers method at a load of 2.942 N, in accordance with the ISO 6507-1 [28] standard using the Future-Tech FM-ARS 9000 hardness tester with an automatic measurement line and an image analysis system (Future-Tech Corp., Kawasaki, Japan). Measurements were performed on the cross-sectional surface according to Figure 4.

## 4. Results and Discussion

### 4.1. Operational Tests

**Figure 5:**
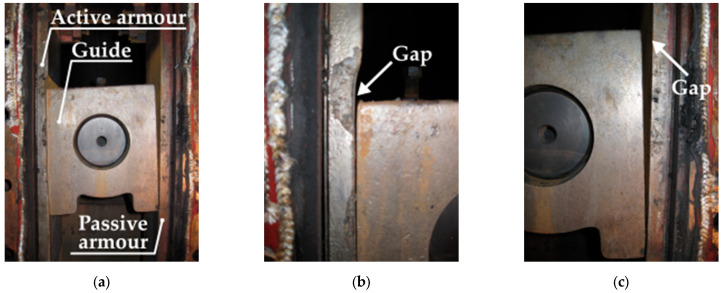
Guidance system with thermal sprayed coatings after 1578 h of operation: (**a**) view of the entire system, (**b**) view of the gap between the guide and the active armour, and (**c**) view of the gap between the guide and the passive armour.

**Figure 6:**
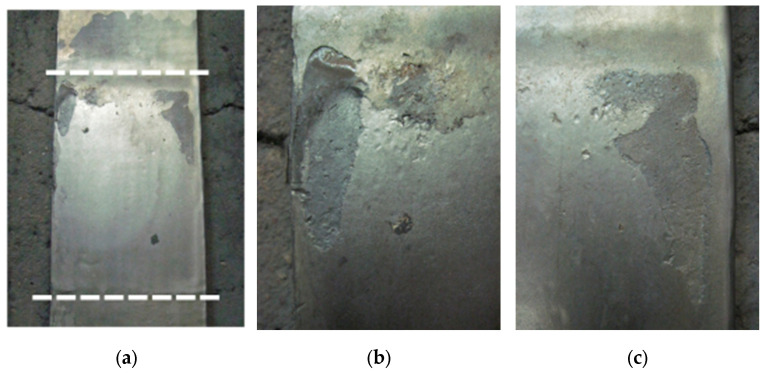
Active armour with a flame-sprayed coating after 1578 h of operation: (**a**) view of the coating with the working area of the friction pair marked (white-dashed lines) and (**b**,**c**) view of the coating in the areas with the greatest wear.

The aim of the study was to compare the wear resistance of layers and coatings applied to guides and active armour in real conditions. The results of measurements of the gap width in relation to the operating time of the analysed guide sets and the determined wear resistance are presented in Table 7. A view of the guidance system with sprayed coatings after 1578 h of operation is shown in Figure 5, and the view of the sprayed active armour after disassembly (after 1578 h of operation) is shown in Figure 6.

The guidance system with overlay welded layers was operated for 4540 h, i.e., for the entire assumed test period in real conditions. During this time, in accordance with the technical and operational documentation of the mill manufacturer, five (every 700–800 h of operation) adjustments were made to the width of the construction gap between the guide and the passive armour. The measured gap widths in the upper part of the set were in the range of 2.6–3.8 mm and in the lower part were in the range of 2.4–3.6 mm. For the group of measurements carried out in the upper and lower parts of the set after a given period of operation, the smallest difference was 0.1 mm and the largest was 0.2 mm. There was no hole present in the overlay welded layer of base materials on the guide and active armour. The analysed layers wore out evenly. No further propagation of existing cracks or spalling of the deposited layers was observed. The determined wear resistance of the set was 416–1190 h/mm. The coefficient of variation of wear resistance in the time intervals between subsequent gap measurements was 26.59%, while for five periods (excluding the first period), it was 10.64%. The calculated values of the coefficients of variation may indicate the most common course of the friction pair wear process. In the first period, running-in took place (with a decrease in the wear intensity), resulting from the topography of the surface of the layer with straight beads. As the running-in process progressed, the surface elevations (irregularities) decreased, and thus, the actual contact area increased. As a result, the average unit pressure decreased. After the running-in process, there was a period of stabilized wear with constant wear intensity (wear resistance: 883–1190 h/mm). Most likely, during the operation time of 4540 h, the third wear period (i.e., accelerated wear) did not begin [29].

The set with thermal sprayed coatings was used for 1578 h and was dismantled because after that period the gap between the guide and the passive armour was 11.0 mm, and this could result in an improper operation of the mill. At 748 h after installation, the gap was adjusted, which was 6.7 mm in the upper area and 2.8 mm in the lower area. In both periods of operation (after 748 h and after 1578 h), the sprayed coating wore out unevenly. The sprayed coating was abraded on the analysed elements (Figure 6), which resulted in a significant increase in the intensity of wear. After the overwearing of local coating (holes formed in the base material), the guide was further localized into the active armour, and as a result, the construction gap reached a value of 11.0 mm. Small areas of adhering ground carbon were visible on the surface of the sprayed coatings. The determined wear resistance of the set with sprayed coatings was 159 h/mm for the first period (748 h into the operation from installation) and 92 h/mm for the second period (another 830 h after installation). In both analogous periods, the wear resistance of the sprayed guidance system was much lower than that of the overlay welded system. In the study [7], thermal sprayed coatings with the composition of a nickel alloy with fine borides and carbides also showed a lower abrasion resistance than the hardfacing layer with the structure of high-alloy chrome cast iron.

### 4.2. Macro- and Microstructure Studies

**Figure 7:**
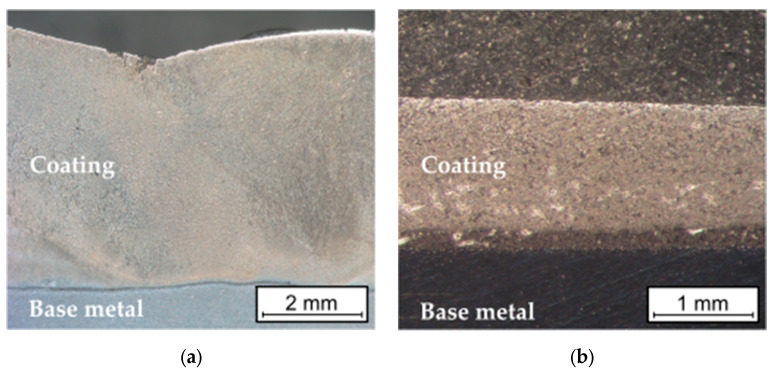
Macrostructure of (**a**) self-shielded flux cored arc surfacing layer and (**b**) flame-sprayed coating.

The metallographic tests carried out did not reveal any defects in the applied layers and coatings, Figure 7. 

**Figure 8:**
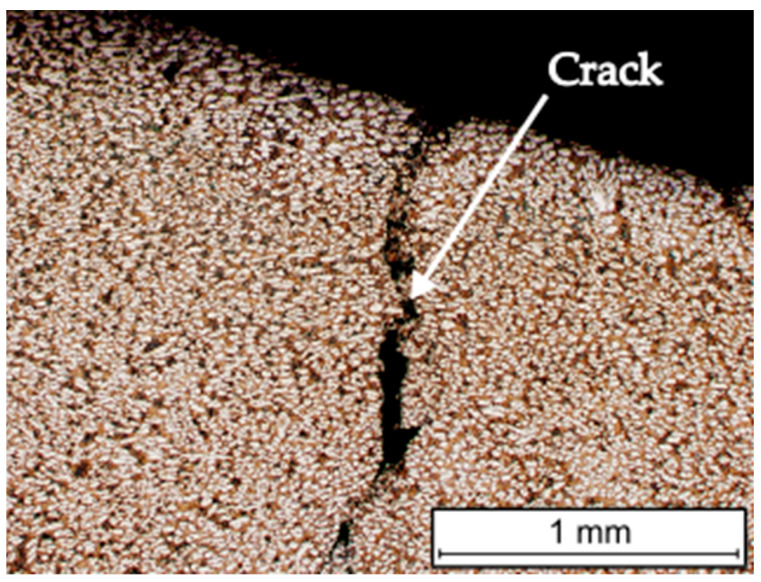
View of a crack in a layer produced by self-shielded flux cored arc surfacing.

The cracks were found in the overlay welded layer (Figure 8), which ran perpendicular to the direction of deposited beads and did not penetrate the base material. These types of cracks (relief-check cracks) enable the relaxation of stresses arising from the solidification and cooling of the layer during the overlay welding process. In surfaced layers with chromium cast iron alloys intended for operation in abrasive wear conditions with low-impact loads, cracks are permissible if they do not penetrate the base material, are not oriented parallel to the fusion surface, and do not lead to the detachment of fragments of the welded layer [30,31,32]. In tribological tests, where abrasion occurs under high pressures or significant impact loads, the uneven wear of the weld deposits could occur as a result of fragments being chipped off [9,30]. No cracks were observed in the sprayed coatings. Both overlay welded layers and thermal sprayed coatings are properly bonded to the substrate material. The sprayed coating was completely melted, which proves that the melting process was carried out correctly [22,33]. There was no melting of the surface of the base material of samples flame-sprayed with the powder. The coating had a homogenous and non-lamellar structure, which is characteristic of sprayed coatings [34].

**Figure 9:**
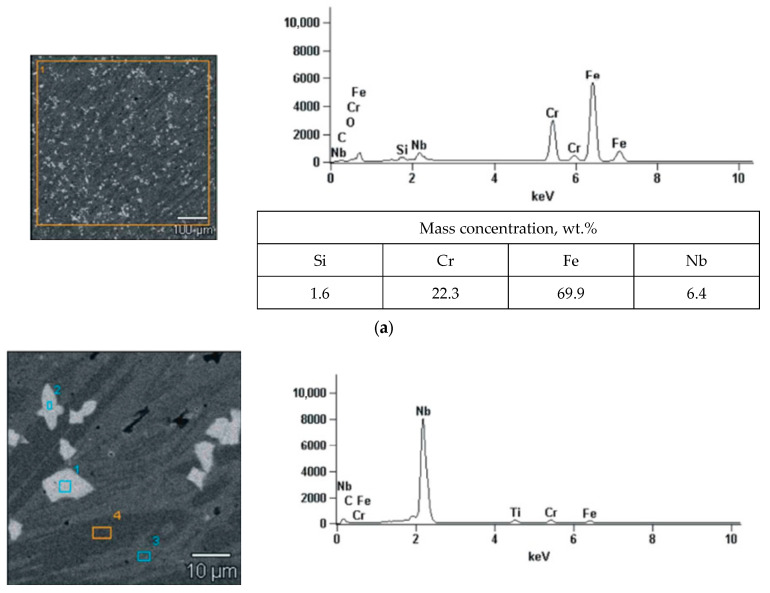

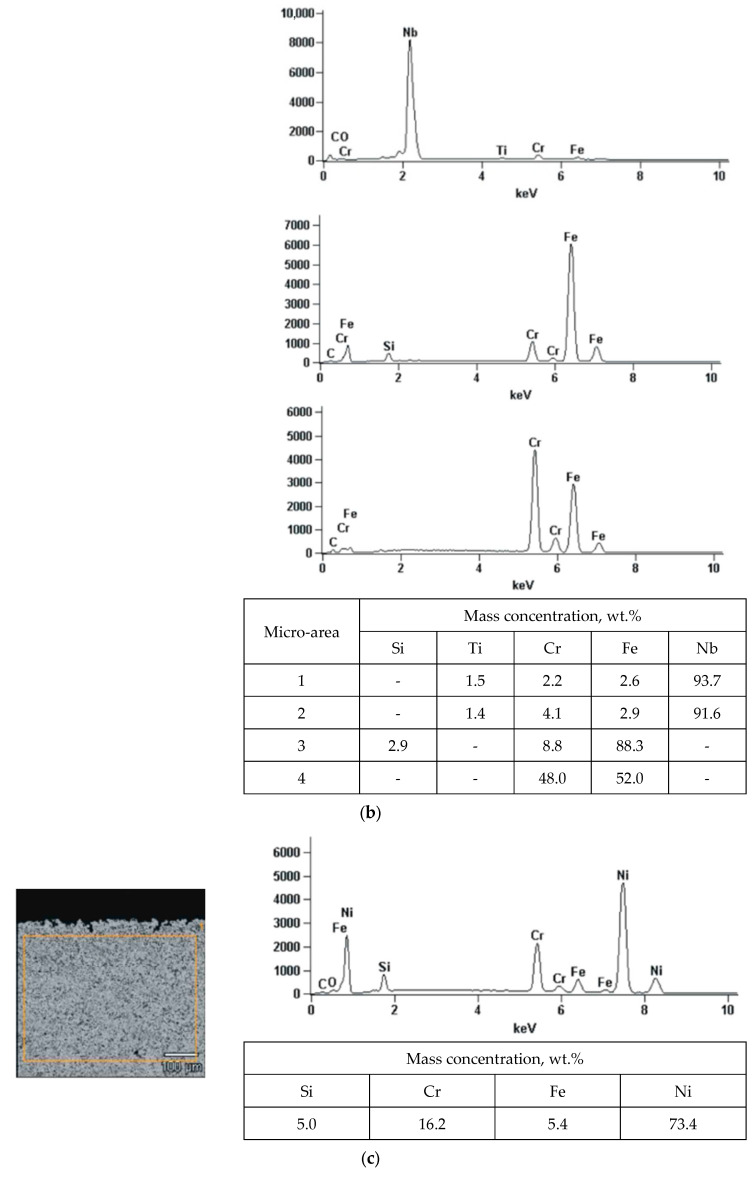

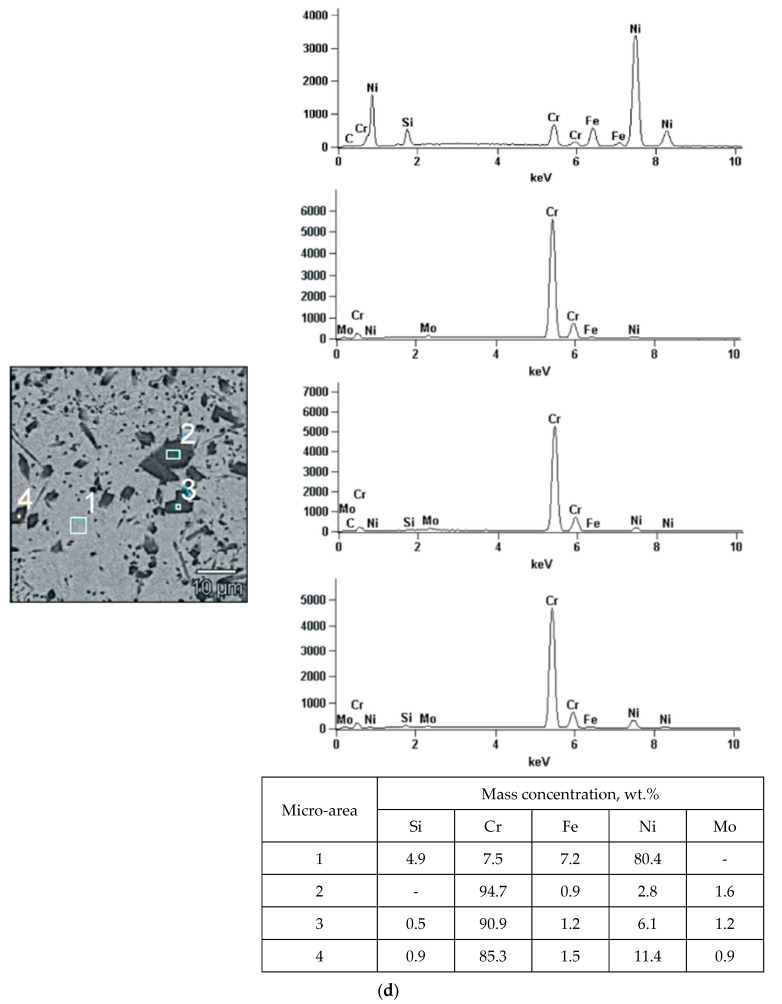
The examined micro-area along with the results of the analysis of the chemical composition of EDS: (**a**,**b**) self-shielded flux cored arc surfacing layer and (**c**,**d**) flame-sprayed coating.

**Figure 10:**
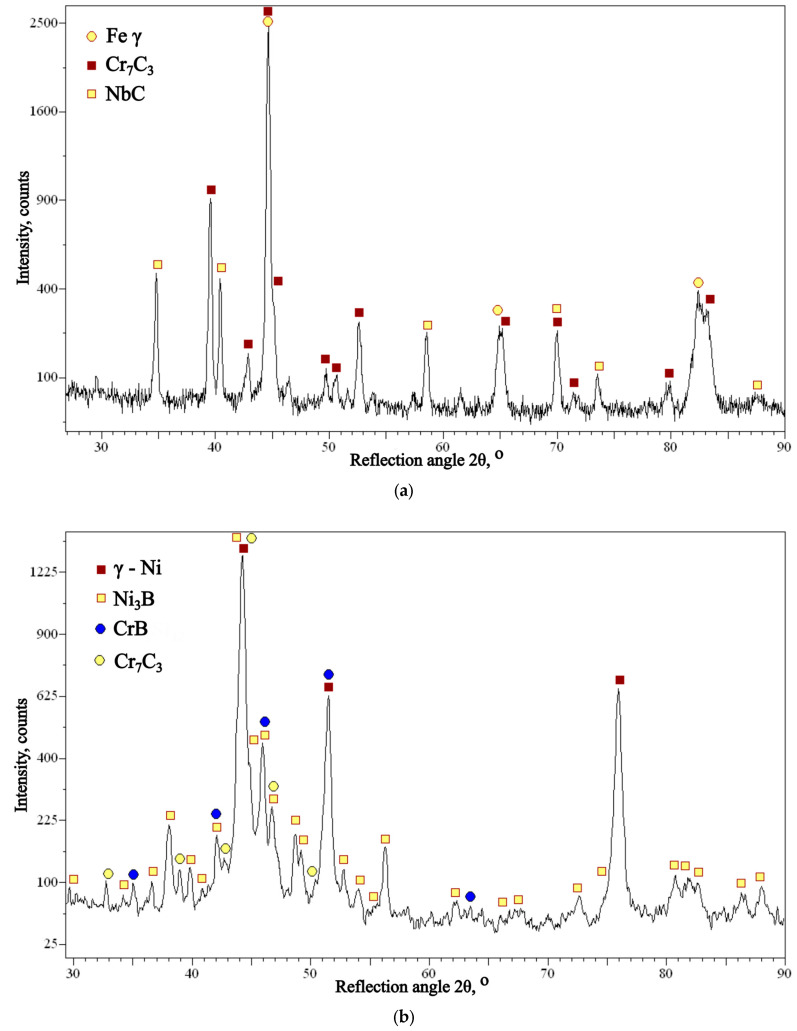
X-ray diffractogram of (**a**) self-shielded flux cored arc surfacing layer and (**b**) flame-sprayed coating.

**Figure 11:**
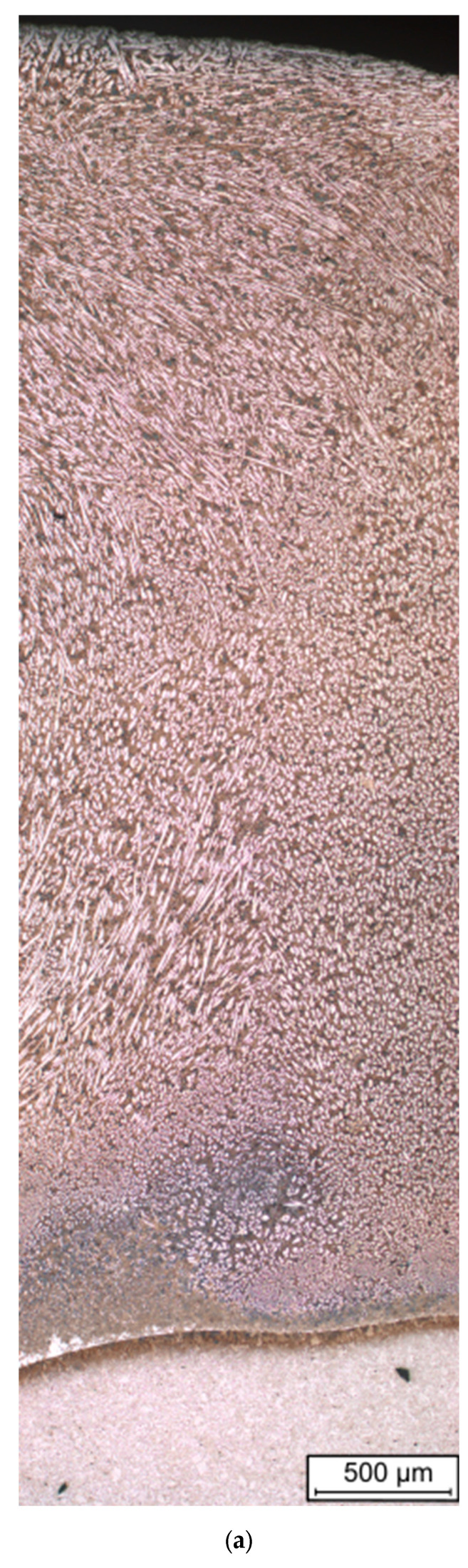

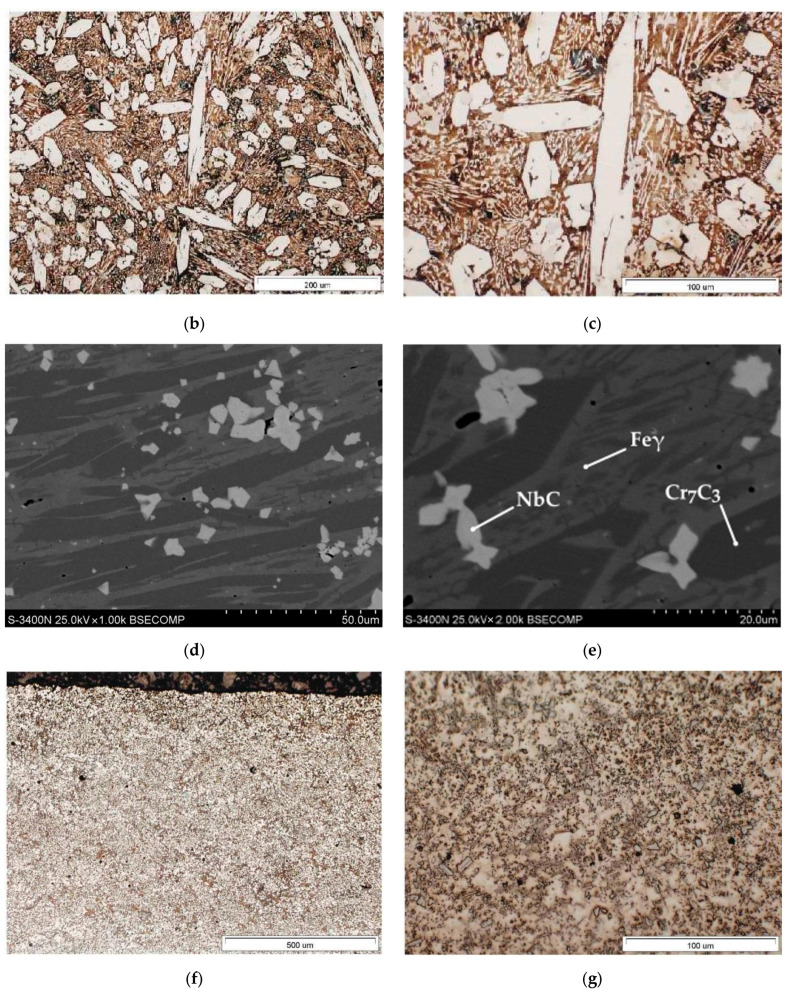

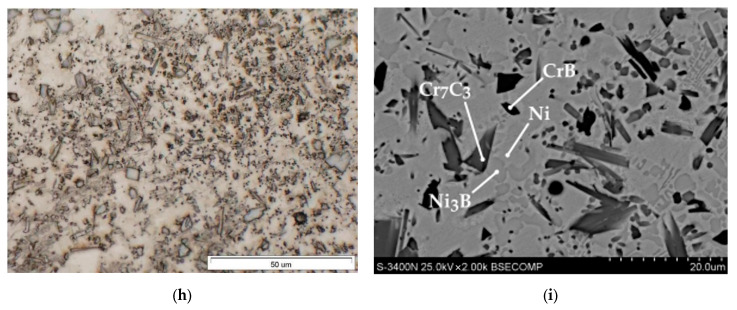
Microstructure of (**a**–**e**) self-shielded flux cored arc surfacing layer and (**f**–**i**) flame-sprayed coating.

Based on the analysis of the chemical composition (Figure 9) and the identification of the phase composition (Figure 10), as well as microscopic metallographic examinations (Figure 11), it was possible to conclude that the overlay weld had a structure composed of larger Cr_7_C_3_ carbides and smaller NbC carbides in an austenite matrix. The sprayed coating consisted of CrB borides and Cr_7_C_3_ carbides in a matrix of γ-Ni solid solution and Ni_3_B. According to Kim et al. [35], melting the sprayed Ni-Cr-B-Si-C coating results in obtaining CrB and Cr_7_C_3_. The analysis of the chemical composition in micro-areas (Figure 9) indicated the occurrence of Si mainly in the matrix of the γ-Ni solid solution.

The porosity of the thermal sprayed coating, determined as the arithmetic mean of ten measurements based on the analysis of ten microstructure images, is 1.08%. Self-fluxing coatings after melting are characterized by a lower porosity [35]; hence, the very low value of the determined porosity most likely results from the melting of the coating after spraying.

### 4.3. Hardness Measurements

The hardness of the overlay welding layer measured on the cross-sectional surface was within the range of 837–1061 HV0.3, and that of the sprayed coating was within the range of 579–714 HV0.3 (Table 8). The hardness measurements showed a very high average hardness of the overlay welding layer (932 HV0.3) and a relatively high average hardness of the sprayed coating (637 HV0.3), respectively. The range of hardness measurements for the sprayed coating was smaller (135 HV0.3) than that of the overlay welded layer (230 HV0.3).

## 5. Conclusions

Based on the study conducted for the comparison of overlay welded layers and flame-sprayed coatings in terms of the elements of the retaining ring guidance system in a ball-race mill for coal grinding, the conclusions that can be drawn are as follows:

1. In operational tests, the wear resistance of self-shielded flux cored arc surfacing layers with the structure of high-alloy chromium cast iron is much greater than that of coatings flame-sprayed with nickel-based powder. For comparable periods of use, this resistance is 416 and 922 h/mm for overlay welded layers and 159 and 92 h/mm for thermal sprayed coatings.
2. The layers deposited by flux cored arc surfacing wore out evenly, and there was no propagation of existing cracks or spalling of the layer. No hole in the surfacing layer of the base material was found after 4540 h of use. The thermal sprayed coating wore out locally (the exposure of the base material) in up to 748 h. This resulted in an increased level of wear.

## Figures and Tables

**Table 1:** Chemical composition of materials for guides and active armour according to the manufacturer (Huta Stalowa Wola S.A., Stalowa Wola, Poland).

Element	Material	Chemical Composition, wt.%										
		C	Si	Mn	P	S	Cr	Mo	Ni	V	Cu	N
Active Armor	S235JR	0.15	0.24	1.15	0.022	0.007	0.01	-	0.01	0.001	0.01	0.003
Guide	GS240	0.19	0.43	1.12	0.015	0.012	0.09	0.01	0.04	0.001	0.06	- ^1^

Note: ^1^ lack of data.

**Table 2:** Mechanical properties of materials for guides and active armour according to the manufacturer (Huta Stalowa Wola S.A., Stalowa Wola, Poland).

Element	Material	Yield Strength, MPa	Tensile Strength, MPa	Elongation, %
Active Armor	S235JR	285	468	31
Guide	GS240	307	491	23

**Table 3:** Chemical composition and hardness of the weld deposit of the self-shielded flux cored wire SK A43-O according to the manufacturer (Voestalpine AG, Linz, Austria).

Chemical Composition, wt.%						Hardness, HRC
C	Cr	Nb	Si	Mn	Fe	
5.6	20.2	6.7	1.3	0.2	bal.	64

**Table 4:** Chemical composition and hardness of the powder Oerlikon Metco 15E according to the manufacturer (OC Oerlikon Corporation AG, Pfäffikon, Schwyz, Switzerland).

Chemical Composition, wt.%						Hardness, HRC
Cr	B	Si	C	Fe	Ni	
17.0	3.5	4.0	1.0	4.0	bal.	60

**Table 5:** Parameters of surfacing layers with SK A43-O self-shielded flux cored wire.

Wire Diameter, mm	Welding Current, A	Arc Voltage, V	Electrode Extension, mm	Travel Speed, cm/min
2.4	280	28	35	50

Note: the overlay welds were surfacing automatically, with stringer beads parallel to the long side of the guide. Two layers were deposited for operational tests.

**Table 6:** Parameters of flame spraying of coatings with Metco 15E powder.

Oxygen Pressure, Bar	Acetylene Pressure, Bar	Air Pressure, Bar	Number of the Orifice for the Powder
4.0	0.7	1	4

Note: standard modular nozzles regulating the flame outlet (SSM 20) were used. Slightly carburizing flame was used. The spraying distance was 200 mm. The surface of the base material for spraying was prepared with electrocorundum abrasive blasting. Before spraying, the surface of the substrate was heated to 280 °C. After heating, a thin powder coating was applied to protect the surface against oxidation, and then, a coating with a thickness of approx. 1.0 mm was applied. The coating was melted using a normal flame.

**Table 7:** Results of wear resistance tests of overlay welded and thermal sprayed guides and active armour in operational conditions.

Working Time of Guide Sets (to Their Mounting), h	Width of the Gap between the Guide and Passive Armor, mm				Remarks		Wear Resistance, h/mm ^1^	
	Set of Overlay Welded Parts		Set of Thermal Sprayed Parts		Set of Overlay Welded Parts	Set of Thermal Sprayed Parts	Set of Overlay Welded Parts	Set of Thermal Sprayed Parts
	Dimension “a” (Upper Part of the Set), mm	Dimension “b” (Bottom Part of the Set), mm	Dimension “a” (Upper Part of the Set), mm	Dimension “b” (Bottom Part of the Set), mm				
0	2.0	2.0	2.0	2.0	Assembly	Assembly	-	-
748	3.8	3.6	6.7	2.8	Gap adjustment	Gap adjustment	416	159
1578	2.9	2.7	11.0	2.5	Gap adjustment	Removal	922	92
2313	2.7	2.6	-	-	Gap adjustment	-	1050	-
3120	2.8	2.7	-	-	Gap adjustment	-	1009	-
3834	2.6	2.4	-	-	Gap adjustment	-	1190	-
4540	2.8	2.7	-	-	Removal	-	883	-

Note: ^1^ wear resistance is defined as the operating time (in hours) per unit of wear (corresponding to the size of the structural gap between the guide and the passive armour).

**Table 8:** Results of HV0.3 hardness measurements on the cross-sectional surface of the overlay welded layer and the sprayed coating.

Method	Filler Metal	Hardness Measurement Point HV0.3 According to Figure 4				
		1	2	3	4	5
Self-shielded flux cored arc surfacing	SK A43-O	928	965	1067	864	837
Flame spraying	Metco 15E	634	616	579	640	714

## Data Availability

Research data cannot be made available.
